# Incidence of ligamentous laxity and scoliosis among the population of the United Arab Emirates

**DOI:** 10.1186/1748-7161-7-S1-O70

**Published:** 2012-01-27

**Authors:** K Bagnall, H Al Hamsi, A Al Kaabi, T Al Mazrouee, A D’souza

**Affiliations:** 1 UAE University, Al Ain, United Arab Emirates

## Background

Among a general lack of awareness of scoliosis, the incidence of Adolescent Idiopathic Scoliosis (AIS) in the United Arab Emirates is not known although efforts are currently being made to remedy this. The Emirati people have a unique and well-structured culture with strong family relationships and might be considered a closed group. Consequently, there are some factors within this group that suggest that the incidence of AIS might be higher than in other parts of the world. In this study, the incidence of ligamentous laxity among young Emirati women was measured because this has been associated many times previously with AIS and casual observation has suggested that ligamentous laxity is prevalent among many young Emirati women.

## Material and methods

The degree of extension of the middle metacarpo-proximal-phalanx joint and thumb abduction were measured on both hands using standard, previously reported techniques of 100 randomly-selected, young Emirati women. The results were compared with similar published data from the United Kingdom.

## Results

The results showed that there was a higher incidence of ligamentous laxity among the emirati population in both the degree of extension of the middle metacarpo-proximal-phalanx joint as well as thumb abduction when compared to the values from the UK.

## Conclusions

These results suggest that the incidence of AIS in the UAE (when determined) might be expected to be greater than in other parts of the world due to a higher incidence of ligamentous laxity among the susceptible population.

